# Quality of life measured by OHIP-14 and GOHAI in elderly people from Bialystok, north-east Poland

**DOI:** 10.1186/1472-6831-14-106

**Published:** 2014-08-20

**Authors:** Ewa Rodakowska, Karolina Mierzyńska, Joanna Bagińska, Jacek Jamiołkowski

**Affiliations:** 1 Department of Restorative Dentistry, Medical University of Bialystok, Bialystok, Poland; 2 NZOZ Przychodnia Stomatologiczna Lucyna Mierzyńska-Ładny dentine Stomatologia, Bialystok, Poland; 3 Department of Dentistry Propaedeutics, Medical University of Bialystok, Bialystok, Poland; 4 Department of Public Health, Medical University of Bialystok, Bialystok, Poland

**Keywords:** Oral health-related quality of life, OHIP-14, GOHAI, Quality of life, Elderly, Poland, “Fatigue effect”

## Abstract

**Background:**

The Oral Health Impact Profile-14 (OHIP-14) and the Geriatric/General Oral Health Assessment Index (GOHAI) have never been compared for a group of the same subjects in the Polish population. The aim of the study was to compare the OHIP-14 and GOHAI measures.

**Methods:**

178 independently living people over the age of 55 were included in the study. The GOHAI and OHIP-14 measures were used. Other variables included age, gender, self-ratings of oral general health, education, number of missing teeth, chewing problems and dry mouth.

**Results:**

The mean age of respondents was 70.8 years. The internal reliability (Cronbach’s alpha) showed a high internal consistency for both measures. Spearman’s rank correlation coefficient between the GOHAI and OHIP-14 scores was 0.81. Using the additive method of creating scores, 1.1% of respondents had the GOHAI score of zero, indicating no impact from oral conditions, while 13.5% of them had an OHIP-14 score of zero. Dental status, partial dentures, chewing problems, dry mouth and self-rated oral health were significantly associated with the results of the GOHAI and the OHIP-14 (Kruskal–Wallis test, Mann–Whitney U test). The numbers of preserved and missing teeth significantly correlated with the GOHAI and the OHIP-14, while DMF was significantly associated with the GOHAI only. 6 individuals with discrepant results were revealed. After the exclusion of the abovementioned patients, the internal reliability (Cronbach’s alpha) still showed a high internal consistency, and the correlation between the GOHAI and OHIP-14 scores using Spearman’s rank-correlation coefficient increased to 0.87. This phenomenon was identified as a “fatigue effect”.

**Conclusions:**

There was a strong correlation between the GOHAI and the OHIP-14. Both instruments demonstrated good discriminant properties and helped capture the respondents’ oral health problems. The questionnaires should be randomly distributed to avoid the influence of “fatigue effect” on the results of a comparison of different measures.

## Background

The population of people worldwide is constantly growing older and their health-related quality of life (HRQoL) is an increasing public health concern [1]. The relation between oral health and general health is particularly visible among old people because the large proportion of them does not or even cannot follow the necessary teeth and denture hygiene practices, which has additional negative oral health impacts [2,3]. The outcomes of oral health conditions and therapy for those conditions are described by the term ‘oral health-related quality of life’ (OHRQoL) [4,5]. This concept refers to the extent to which the oral diseases impact on individuals’ normal functioning and is regarded as an integral part of general health and well-being. OHRQoL is recognized by the WHO as an important part of the Global Oral Health Program [6,7]. This is a multidimensional concept that deals with the quality of life (QoL) related to oral health and diseases [7-9]. It has been widely used in theoretical and practical fields including dental research, clinical trials and other studies evaluating the outcomes of preventive and therapeutic programs.

The Oral Health Impact Profile (OHIP) and the Geriatric/General Oral Health Assessment Index (GOHAI) are regarded as the most comprehensive assessments for measuring the OHRQoL [4,10]. They have been widely used in research studies on various populations. The measures differ in terms of the item content. The OHIP-14 is a shorter version of the OHIP-49 described by Slade and Spencer but it retains the original conceptual dimensions contained in the OHIP-49 [11,12]. Its aim is to assess seven dimensions of impacts of oral conditions on people’s OHRQoL including functional limitation, physical pain, psychological discomfort, physical disability, psychological disability, social disability and handicap [11]. It refers to the period of one year. According to Locker et al. [13], OHIP-14 is patient-centered, gives a greater weight to psychological and behavioral outcomes, is better at detecting psychosocial impacts among individuals and groups, and better meets the main criteria for the measurement of OHRQoL.

The GOHAI measure is a 12-item questionnaire originally developed in 1990 in the USA for use with elderly populations with three months’ time reference [14]. Lately, it has been also used with younger adult populations, which is reflected in the interchangeable us of the names Geriatric or General Oral Health Assessment Index [15,16]. It was developed to evaluate three dimensions of oral-health related quality of life including physical functions like eating, speech, swallowing; psychosocial functions like worry, concern about oral health, dissatisfaction with appearance, self-consciousness about oral health, avoidance of social contacts because of oral problems; pain or discomfort including the use of medication or discomfort from the mouth [14]. According to Locker et al. [13], the GOHAI gives a greater weight to functional limitations or pain and discomfort. According to the research of Hassel et al., the GOHAI seems to be more appropriate when focusing on subjective oral health with minor clinical changes and immediate clinical aspects [13,17].

As the OHIP-14 gives a greater weight to psychological and behavioral outcomes, and the GOHAI to functional limitations or pain and discomfort both describe different aspects of OHRQoL. No comparison study to explore the ability of these two scales has ever been done in Poland. The aim of the study was to compare the Oral Health Impact Profile-14 (OHIP-14) and the Geriatric/General Oral Health Assessment Index (GOHAI) measures and to assess which instrument was more adequate in Polish subjects. Due to the planned surveys on patients with different disorders our idea was to identify which measure could be more useful in adults in Poland.

## Methods

### Study population

Participants were recruited by means of the convenience sample in a public dental clinic in Bialystok, the biggest city in the north-east part of Poland. The public dental clinic was situated in the center of the city with a good access to public transport. The enrollment period was six months (Oct. 2011 March 2012). The main inclusion criterion was the age of 55 and over. The sample size was based on an a priori assumed correlation between the GOHAI and OHIP scales [13,18,19]. The calculations were performed for following set of hypotheses:

H_0_: ρ_0_ = 0.7 and H_1_: ρ_1_ = 0.8

The type I error probability (α) was set at 0.05 level and type II error probability (β) was set at 0.1 level. For these assumptions the minimal sample size was 162 [20]. We anticipated 10% dropout and therefore the projected sample size was set at 179. Prior to the beginning of the research, the informed consent and the data collection method were approved by the Ethical Committee of the Medical University of Bialystok, Poland. The participation in the study was both anonymous and voluntary and started after a written consent of the participants. The patients were invited to fill the questionnaire during their regular dental checkup or when they had a requested treatment. The patients who were unable to comprehend the questionnaire were excluded from the study to avoid unreliable answers. Data regarding the number of teeth and the caries experience (DMFT) were collected by means of the available dental records.

### Measures

First we focused on the translation and linguistic adaptation of the questionnaires into Polish. We followed the guidelines presented in papers regarding the translation issues in OHRQoL [21-24]. The versions translated by professionals were compared by a review committee, the authors, who are from different fields of dentistry and after minor corrections the final version was created. The questionnaire used in the survey contained both the GOHAI [14] and the OHIP-14 [11] scales. Three months’ reference for the GOHAI and one year’s for the OHIP were used. The response format for both on a Likert-type frequency scale was as follows: very often = 4, fairly often = 3, occasionally = 2, hardly ever = 1, never = 0 [12]. The two measures were compared in terms of their item content. The answers for both instruments were the same. The additive method was used to calculate the GOHAI and the OHIP-14 scores. For the OHIP-14, they were obtained by summing the response codes of the 14 items constituting the measure. Additive scores for the GOHAI were obtained by summing the response codes for the 12 items. Questions were worded positively and negatively to require the participants to consider the answers. The coding of three items like “able to swallow comfortably”, “able to eat without discomfort”, pleased with the look with teeth” were reversed (high score in the GOHAI indicated a low impairment). Consequently, the GOHAI scale ranged from 0 to 48 and the OHIP-14 scale from 0 to 56 with higher scores indicating a poorer oral health-related quality of life. Other questions referred to age, gender, self-rating of oral general health, education and number of missing (M) teeth.

### Data analysis

The Kruskal–Wallis test and the Mann–Whitney’s U test were used to compare the GOHAI and the OHIP-14 scores in relation to self-ratings of oral health, education, chewing ability and dry mouth. The GOHAI and the OHIP-14 scores were dichotomized using median splits. To calculate odds ratios which provided interpretable measure of the strength of the associations between dependent and independent variables, 95% confidence intervals were calculated for odds ratios to verify statistical hypotheses that OR ≠ 1. Spearman’s rank correlation coefficients were used to measure inter-item and item-score correlations as well as correlations of the GOHAI or OHIP-14 scores with age and number of preserved teeth. The values of Cronbach’s alpha were calculated to assess the internal consistency for the whole score and for particular items removed. The statistical analysis was performed using the IBM SPSS Statistics 20.0 software. Statistical hypotheses were verified with a significance level of 0.05.

## Results

Altogether, the final sample consisted of 178 independently living people, 79 men and 99 women. The sample constituted 0.23% of the inhabitants aged 55 and over living in Bialystok [25]. They ranged in age from 55 to 93, were 55.5% were aged 70 and over and the mean age was 70.8 (SD 7.6) years. As for the educational background, only 16.3% of subjects declared higher education, but the majority had secondary education (56.2%). Furthermore, 66.3% of the participants were dentate. Only 5.1% of respondents had more than 20 teeth. The mean number of teeth was 6.2. Among the dentate subjects, 89.0% wore partial dentures, more women (90.5%) than men (87.2%). The internal reliability (Cronbach’s alpha) was 0.89 for the GOHAI and 0.97 for the OHIP-14, showing a high internal consistency. Internal reliability of scales for each single item removed varied between 0.873 (item 11) and 0.896 (item 8) for the GOHAI and 0.954 (item 9,11) and 0. 957 (item 2) for the OHIP-14. The correlation between the GOHAI and OHIP-14 scores using Spearman’s rank-correlation coefficient was 0.81 (p < 0.001). Spearman’s correlations between scales and their items varied between 0.416 (item 8) and 0.668 (item 3) for the GOHAI and 0.690 (item 8) and 0.807 (item 11) for the OHIP-14.

Table 1 shows the percentage of participants who responded very often, fairly often, occasionally or hardly ever to each GOHAI and OHIP-14 item. Four sub-scales were created for each measure using the domains and items listed in Table 1. Using GOHAI ADD scores, 8.4% reported no functional limitations, 6.7% no pain or discomfort, 9.6% no psychological impacts and 19.7% no behavioral impacts. The corresponding statistics when OHIP-14 ADD scores were used were 27%, 23%, 21.3% and 27.5%. In total, for the GOHAI, the percentage answering in the affirmative to each item ranged from 55.1% to 85.4%; for the OHIP-14 these values ranged from 51.1% to 73.0%. Taking into consideration both measures, at least 51.1% of participants answered in the affirmative to each item. None of the subjects scored the maximum in either measure. The GOHAI score ranged from 0 to 45 and the OHIP-14 score ranged from 0 to 48. We found that only 6 participants did not report any issues on the GOHAI scale, and 40 on the OHIP-14 scale. A greater impairment in the OHIP-14 reflected a greater impairment in the GOHAI except scores 0–3 in OHIP-14 where the GOHAI scores reflect 0–37.

**Table 1:** Percentage of subjects responding sometimes, fairly often, very often or all the time to each GOHAI and OHIP-14 item

**GOHAI**	**%**	**OHIP-14**	**%**
*Functional limitation*	*91.6*		*73*
Trouble biting/chewing food	85.4	Trouble pronouncing words	65.7
Uncomfortable to swallow	66.3	Sense of taste worse	67.4
Prevented from speaking	69.7		
*Pain and discomfort*	*93.3*		*77*
Discomfort when eating	75.8	Painful aching in mouth	56.2
Use medication to relieve pain	62.4	Uncomfortable to eat foods	73.0
Teeth, gums, sensitive to hot/cold	75.3		
*Psychological impacts*	*90.4*		*78.7*
Unhappy with appearance	75.3	Been self-conscious	68.5
Worried or concerned	82.6	Felt tense	66.3
Nervous or self-conscious	73.0	Difficult to relax	62.9
Uncomfortable eating in front of people	70.2	Been embarrassed	65.2
		Felt life is less satisfying	68.5
*Behavioral impacts*	*80.3*		*72.5*
Limit kinds or amounts of food	77.0	Diet has been unsatisfactory	62.9
Limit contact with others	55.1	Had to interrupt meals	51.1
		Been irritable with others	61.2
		Difficulty doing usual jobs	53.9
		Totally unable to function	51.1

Using the additive method of creating scores, 1.1% had the GOHAI score of 0, indicating no impact from oral conditions, while 13.5% had an OHIP-14 score of 0 (Table 2). The skewness was 0.09 for the GOHAI and 0.39 for the OHIP-14, with the OHIP-14 scores being more skewed than GOHAI scores. Differences in the distributions of the GOHAI and the OHIP-14 scores were also reflected in their median values of 19.5 (lower quartile 10; upper quartile 26) and 14.5 (lower quartile 4; upper quartile 28), respectively.

**Table 2:** Descriptive statistics: GOHAI and OHIP-14

	**GOHAI ADD**	**OHIP-14 ADD**
Range	0-45	0-48
% with score of 0	1.1	13.5
Mean (SD)	18.9 (10.3)	17.6 (14.3)
Median	19.5	14.5
Skewness	0.09	0.39

ADD- additive method.

**Figure 1:**
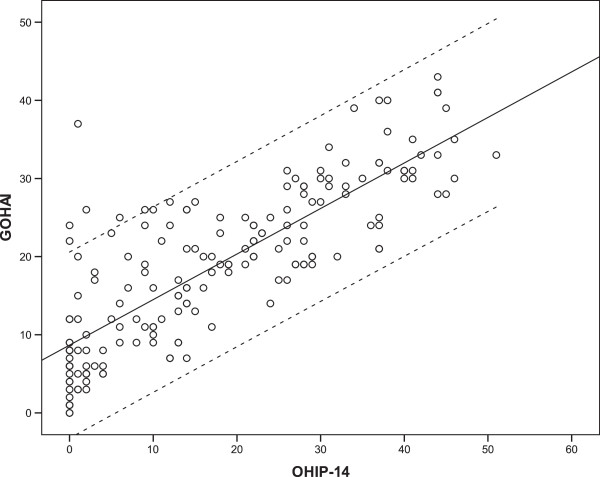
Scatterplot of GOHAI scores vs. OHIP-14 scores with linear regression line and 95% prediction interval.

Table 3 shows mean values of the GOHAI and OHIP-14 and particular grouping variables. Both measures showed significant associations with being dentate and edentulous, self-rated chewing ability, perception of dry mouth and self-rated general health. Gender, education, number of missing teeth and partial dentures did not show any significant relation to the GOHAI and OHIP-14 scores. The odds ratios are shown in Table 4. Dental status, partial dentures, chewing problems, dry mouth and self-rated oral health were significantly associated with the GOHAI and the OHIP-14. For example, participants rating their chewing problems had a 15.6 times greater risk of having the GOHAI score above the median than those without chewing problems, whereas the risk of having the OHIP-14 score above the median was 6.56. The numbers of preserved teeth and missing teeth were significantly related to the GOHAI and the OHIP-14, while DMF (decayed, missing and filled teeth) was only positively associated with the GOHAI. However, the relationship between the age and the OHIP-14 and the GOHAI score measured by Spearman’s rank-correlation coefficients was not found (Table 5).Figure 1 depicts the scatterplot of the GOHAI scores vs. OHIP-14 scores with a linear regression line and a 95% prediction interval. The GOHAI and OHIP-14 scales were highly correlated, but there were six individuals with discrepant results. They were identified as cases outside the 95% prediction interval for the linear relationship between the OHIP-14 and the GOHAI. All subjects were females characterized by high GOHAI and low OHIP-14 scores. After the exclusion of the abovementioned patients, the internal reliability (Cronbach’s alpha) still showed a high internal consistency, however it increased for the GOHAI to 0.97 and decreased for OHIP-14 to 0.9. The correlation between the GOHAI and OHIP-14 scores using Spearman’s rank-correlation coefficient increased to 0.87.

**Table 3:** Mean values of the GOHAI and the OHIP-14 scores of grouping variables

	**GOHAI Mean (SD)**	**OHIP – 14 Mean (SD)**
*Gender*		
Male (n = 79)	18.0 (9.9)	13.0 (13.7)
Female (n = 99)	20.0 (10.4)	17.0 (14.7)
p	0.067	0.268
*School education*		
Primary	23.0 (10.7)	14.5 (14.7)
Secondary	18.0 (10.2)	14.0 (14.4)
Tertiary	21.0 (9.03)	15.0 (13.5)
p	0.082	0.816
*Dental status*		
Dentate (n = 118)	18.5 (9.7)	12.5 (13.0)
Edentulous (n = 60)	23.5 (10.5)	26.0 (15.2)
p	0.002	0.001
*Number of M teeth*		
<=20 (n = 63)	17.0 (9.1)	11.0 (11.9)
>20 (n = 56)	19.5 (10.5)	15.5 (13.9)
p	0.132	0.117
*Partial denture*		
Yes (n = 105)	18.0 (9.5)	12.0 (12.9)
No (n = 14)	24.0 (10.9)	22.5 (12.9)
p	0.064	0.061
*Chewing ability*		
No (n = 87)	11.0 (7.8)	7.0 (10.5)
Yes (n = 91)	25.0 (8.1)	27.0 (13.8)
p	0.000	0.000
*Dry mouth*		
No (n = 67)	17.0 (9.1)	12.0 (11.8)
Yes (n = 111)	21.0 (10.4)	22.0 (15.1)
p	0.002	0.009
*Self-rated oral health*		
Yes (n = 98)	14.0 (8.8)	12.5 (12.4)
No (n = 80)	24.0 (9.5)	22.0 (15.1)
p	0.000	0.000

**Table 4:** Odds ratios

	**GOHAI ADD**	**CI 95%**	**OHIP-14 ADD**	**CI 95%**
Dental status (edentulous vs. dentate)	2.04*	1.082-3.852	2.27*	1.198-4.299
Number of missing teeth (>20 vs. 20 or less)	1.69	0.891-3.209	1.88	0.989-3.585
Partial denture (yes vs. no)	0.49*	0.267-0.904	0.45*	0.241-0.823
Chewing problems (yes vs. no)	15.60*	7.502-32.456	6.56*	3.412-12.623
Dry mouth (yes vs. no)	2.07*	1.115-3.841	2.07*	1.115-3.841
Self-rated oral health (good vs. bad)	0.22*	0.115-0.411	0.40*	0.216-0.728
Gender (females vs. males)	1.509	0.832-2.734	1.38	0.760-2.491

*95% Cl does not include 1; ADD – additive method.

Results of the analyses using dichotomized GOHAI and OHIP-14 scores.

**Table 5:** Spearman’s correlation coefficient between GOHAI and OHIP-14 scores

**Variables**	**GOHAI**		**OHIP-14**	
	**r**	**p**	**r**	**p**
Age (years)	0.145	0.053	0.131	0.080
M (missing teeth)	0.262	0.000	0.267	0.000
DMF (decayed, missing, filled teeth)	0.218	0.003	0.186	0.013
Number of preserved teeth	−0.281	0.000	−0.277	0.000

r – Spearman’s correlation coefficient.

## Discussion

So far, only a limited number of reports comparing the GOHAI and the OHIP-14 among the same, elderly participants living in Canada, Germany, Japan and Lebanon have been published [13,17-19]. Our study is the next and the first conducted in Poland and Eastern Europe. No reports on the GOHAI and the OHIP-14 regarding the Polish population exist as yet and hardly any data about oral health-related quality of life issues in Poland are available in the literature. Both tools are recognized instruments for the evaluation of the oral health-related quality of life in the adults and elderly population in relation to objectively measured oral functions. The GOHAI and OHIP-14 differ in the items content and time references so our idea was to assess which research tool might be more adequate for surveys on OHRQoL in Poland.

The participants of our study were community-dwelling, non-clinical Polish elderly individuals who attended the public dental sector (NHF). With increasing age, more and more Polish people use public dental services. Recent available studies showed that in the 4th quarter of 2010 24% of Polish people at the age of 45–59 years used public dental services, at the age 60–69 - 37.2%, and at the age of 70 and above 45.5% [26]. At the same time, with increasing age, less and less people visit dentists, for example, at the age of 50–59 - 37%, and at the age of 70–79 only 19% [27].

The level of the OHRQoL in the population is expressed by the number of subjects with a score of 0 in the particular measure. A substantial proportion of Polish respondents demonstrated many problems concerning oral health. We found only few subjects with a high OHRQoL: the score of 0 was found only in 1.1% of subjects for the GOHAI and in 13.5% for the OHIP-14. Moreover, these percentages were lowest as compared with other surveys. However, our findings that the number of respondents who scored 0 in the GOHAI was lower than in the OHIP-14 are in accordance with other reports. In Lockers et al., the score of 0 was 8.4% for the GOHAI and 30.0% for the OHIP-14; Hassel et al. obtained 7.1% for the GOHAI and 34% for the OHIP-14. Our results were close to the Japanese study in which 4.6% (the GOHAI) and 12.1% (the OHIP-14) of participants reached the score of 0 [13,17,18].

The likely reason for the low level of OHRQoL in Polish individuals is their bad dental status. It has to be emphasized that, although the mean age of our participants was at least about 70, they had on average only 6.2 teeth. They definitely needed full or partial dentures to sustain the basic oral functions. A threshold of 20 teeth is regarded as a functional and nutritional adequacy of dentition [28]. According to the authors [28,29], tooth loss is strongly associated with the OHRQoL as a more negative impact, and complete or almost complete dentition is associated with the best oral health related quality of life. Our respondents’ dental status was worse than in Lockers et al. study where the participants had on average 7 teeth being ten years older [13]. Furthermore, our data were in contrast to the Japanese study where the mean age of participants was four years less than ours but almost 80% of them had 20 or more teeth [18]. Elderly people from Sweden and Japan reported chewing problems and dry mouth although they had over 20 teeth and hardly any dentures [18,30]. The OHIP scores of 18 in Steel et al. [28] study were much worse for people with fewer teeth than 16 in UK and 25 in Australia. In Brazil, the GOHAI score over 30 was also associated with lost teeth [31].

In Poland, dental problems of older adults and elderly people do not receive the necessary attention, probably because caries in children still is a substantial burden [32,33]. The National Oral Health Survey conducted in Poland in 2009 in the population aged 65–74 years revealed 43.9% of edentulous individuals and the average of 6.6 preserved teeth [33]. In our study we observed that fewer patients were edentulous compared to the national average, but the number of preserved teeth was similar.

The baseline of the problems reported in the surveys on OHRQoL could differ in particular countries due to such factors as the affluence of the society or the educational level. In general the expectations and life experience of people living in developed countries indicate that it is possible to have full dentition in elderly age and avoid dentures [34-37]. The GOHAI and the OHIP-14 emphasize items that assess functional limitations and pain, and those showing psychological and behavioral impacts. “Behavioral impacts” health domain being the least frequently reported in our study in the GOHAI and the OHIP-14 measure is consistent with other studies conducted in Canada and Japan [13,18]. However, our respondents declared such impacts in over 80% in the GOHAI and over 60% in the OHIP-14, which was much more than in the mentioned studies. The differences are visible in the most frequently reported health domains. In Polish respondents, it was “pain and discomfort” in the GOHAI and “psychological impacts” in the OHIP-14, which was similar to the Japanese study [18]. But in the study of Locker et al. “functional limitations” were the most frequently reported items [13].

Our results showed that both, the GOHAI and the OHIP-14 detected the impacts of oral disorders in the evaluated Polish population. However, differences could be observed between the GOHAI and the OHIP-14 in terms of discriminating the oral health-related quality of life outcomes. As a matter of fact, the GOHAI and the OHIP-14 showed a strong correlation in our (0.81) study and in all mentioned Canadian (0.73), Japanese (0.728), German (>0.8) and in Lebanes studies [13,18,19]. According to Hassel et al. [17], in case of assessing a broader concept of the OHRQoL, the OHIP-14 should be chosen. We also proved a higher internal reliability (Cronbach’s alpha) for the OHIP-14 compared to the GOHAI, due to its better internal consistency. This can be partly explained by the fact that the OHIP-14 has more items than the GOHAI and according to Locker et al. [13] it is also a more homogenous measure with the majority of psychosocial outcomes. Studies conducted by Locker et al., Hassel et al., Ikebe et al., and Osta et al. also showed that the GOHAI was more successful in detecting elderly people’s oral health problems [13,17-19]. However, having regard to the fact that the OHIP-14 encompasses a longer time interval than the GOHAI, the opposite proportion might be expected. According to Lockers et al. [13], the GOHAI gives a greater weight to the more immediate outcomes like functional limitations and pain and discomfort, and therefore more common outcomes of oral disorders compared to the OHIP-14 which focuses on more severe and less common, like psychological and behavioral outcomes. In our study the GOHAI showed an impairment that was not reflected by the OHIP-14. Consequently, the studies that assessed only one measure, either the OHIP-14 or the GOHAI, did not show the full spectrum of the problem. In our study, no respondents scored the maximum on either measure showing the ceiling effect, although the results were definitely worse than those obtained by subjects in Canada, Japan and Germany, most likely due to the fact that the majority of participants reported more oral pain, functional and psychological problems [13,17,18].

We observed six cases with discrepant reports of impacts that should be characterized to eliminate bias results. They were all women (6 people) characterized by high GOHAI and low OHIP-14 scores. These respondents were gradually losing interest in the survey which manifested itself in decreasing mean answers to subsequent questions shifting towards 0, which corresponds to the answer ‘never’. The described problem affected the OHIP-14 scale more than the GOHAI and finally resulted in an underestimation of the OHIP-14 scores compared to the GOHAI. Such pattern was not observed in other participants, which indicated a uniform distribution of standard errors. The scores of these respondents might influence the overall results, however, the internal consistency of the GOHAI and the OHIP-14 calculated for both variants of data remained at a high level. As far as we know, the issue of discrepant reports was not discussed in the previous studies so we can only suggest why we encountered subjects with discrepant reports. A probable explanation is that the patients with discrepant reports might lose interest in filling in a questionnaire. This phenomenon should be identified as a “fatigue effect” [38]. We reckon that the way to avoid the influence of the “fatigue effect” on the results is to randomly deliver first GOHAI and then OHIP-14 to a half of the participants than vice versa to another half of the group.

Our study obviously has some limitations. First, the wide range of participants’ age being 55 and over could be considered as a limitation. We decided to set the inclusion criterion at the age of 55 because both measure were recently used in various age groups [15-17]. Another limitation is that the data were gathered by means of a convenience sample from individuals who attended a public dental clinic for a regular dental checkup or had a requested treatment. It is possible that their dental status could be worse compared to non-attending patients, which is why our sample was not representative for the entire Polish population. However, we primarily concentrated on the comparison of these two measures in Polish elderly people. Third, both questionnaires have never been translated before into Polish and validated for the Polish population. Thus, the results may be specific to this study as long as they can be confirmed by another study on a Polish population with a similar age range attending specific dental offices.

## Conclusion

There was a strong correlation between the GOHAI and the OHIP-14. Dental status, chewing ability, dry mouth and self-related oral health in the evaluated Polish elderly group were strongly associated with problems identified using the GOHAI and the OHIP-14. In our study both instruments demonstrated good discriminant properties and helped capture the respondents’ oral health problems. Discrepant reports should be addressed in future studies comparing both scales in order to eliminate bias results. The questionnaires should be randomly distributed to avoid the influence of “fatigue effect” on the results of a comparison of different measures.

## Abbreviations

OHIP-14: The Oral Health Impact Profile-14; GOHAI: the Geriatric Oral Health Assessment Index; HRQoL: Health-related quality of life; OHRQoL: Oral health-related quality of life; ADD: Additive method; r: Spearman’s correlation coefficient; CI: Confidence intervals.

## Competing interests

The authors declare that they have no competing interests.

## Authors’ contributions

ER contributed with concept and design, analysis and interpretation of data, revised critically, responsible for drafting; KM acquisition of data analysis and interpretation of data revised critically; JB contributed with analysis and interpretation of data revised critically; JJ contributed with statistical analysis, analysis and interpretation of data revised critically. All authors read and approved the final version of the manuscript.

## Pre-publication history

The pre-publication history for this paper can be accessed here:

http://www.biomedcentral.com/1472-6831/14/106/prepub
